# NPPC and AREG supplementation in IVM systems alter mRNA translation and decay programs-related gene expression in bovine COC

**DOI:** 10.1590/1984-3143-AR2023-0101

**Published:** 2024-07-08

**Authors:** Helena Fabiana Reis de Almeida Saraiva, Juliano Rodrigues Sangalli, Luana Alves, Juliano Coelho da Silveira, Flávio Vieira Meirelles, Felipe Perecin

**Affiliations:** 1 Faculdade de Zootecnia e Engenharia de Alimentos, Departamento de Medicina Veterinária, Universidade de São Paulo, Pirassununga, SP, Brasil

**Keywords:** *In vitro* maturation, pre-maturation, ZFP36L2, oocyte, cumulus cells

## Abstract

During oocyte meiosis resumption, a coordinated program of transcript translation and decay machinery promotes a remodeling of mRNA stores, which determines the success of the acquisition of competence and early embryo development. We investigated levels of two genes related to mRNA translation (*CPEB1* and *CPEB4*) and two related to mRNA degradation (*CNOT7* and *ZFP36L2*) machinery and found *ZFP36L2* downregulated in *in vitro*-matured bovine oocytes compared to *in vivo* counterparts. Thereafter, we tested the effects of a pre-IVM step with NPPC and a modified IVM with AREG on the modulation of members of mRNA translation and degradation pathways in cumulus cells and oocytes. Our data showed a massive upregulation of genes associated with translational and decay processes in cumulus cells, promoted by NPPC and AREG supplementation, up to 9h of IVM. The oocytes were less affected by NPPC and AREG, and even though ZFP36L2 transcript and protein levels were downregulated at 9 and 19h of IVM, only one (*KDM4C*) from the ten target genes evaluated was differently expressed in these treatments. These data suggest that cumulus cells are more prone to respond to NPPC and AREG supplementation *in vitro*, regarding translational and mRNA decay programs. Given the important nursing role of these cells, further studies could contribute to a better understanding of the impact of these modulators in maternal mRNA modulation and improve IVM outcomes.

## Introduction

During oocyte growth, RNAs and proteins are continuously synthesized and accumulated in order to support development throughout the maternal-to-embryo transition (MET), which starts with meiosis resumption and ends at the embryo genome activation (Bettegowda et al., 2008). The proper functioning of mRNA translation and degradation machinery during this period directly affects oocyte competence to be fertilized and the embryo’s ability to undergo the first cleavages and activate its genome (Gosden and Lee, 2010; Richter, 2007). Since a significant proportion of *in vitro*-produced embryos fail to activate their genome and arrest the development at the 8- to 16-cell stage (Memili et al., 1998), there may be a correlation between the transcript stocks profile in the oocyte and the embryonic ability to progress during development *in vitro*. Transcriptomic analyses of oocytes show that “modulation of translation”, “mRNA degradation”, “protein functions”, “DNA replication” and “cell cycle regulation” are transcript categories associated with higher developmental competence *in vitro* (Mourot et al., 2006; Labrecque et al., 2013; Graf et al., 2014). Therefore, the maternal mRNA stores profile during final growth can mirror the quality and developmental capacity of an oocyte.

Complex, temporal, and orchestrated waves of polyadenylation and deadenylation of maternal mRNAs modulate the oocyte meiotic division (Belloc and Mendez, 2008; Belloc et al., 2008). Signals from the granulosa are responsible for activating, in the oocyte, the translation of key proteins involved in meiotic progression, as well as for the degradation of maternal mRNA (Lefebvre et al., 2002; Zhang et al., 2015). The soma-germ cell interactions during follicle growth direct cytoplasmic and nuclear events that culminate in oocyte acquisition of competence. LH-mediated signals from granulosa determine changes in oocyte chromatin configuration, transcription quiescence, and re-entering in the meiotic cell cycle (Gosden and Lee, 2010). Although this process can occur in oocytes freed from somatic cells, fertilization and early development become compromised (Eppig, 1991; Luciano et al., 2005). These studies demonstrate that adequate cytoplasmic and molecular maturations, i.e the accumulation of mRNA and proteins during the oocyte growth, improve the ability of the oocyte to be fertilized and develop during the transcription-silenced MET. Since *in vitro* maturation (IVM) systems involve the isolation of cumulus-oocyte complexes from the follicles, the loss of granulosa-mediated signaling could affect the mRNA translation and decay pathways, which could result in a failure of the meiotic cell cycle or development arrest during embryo genome activation.

Studies have demonstrated the link between the mRNA translation and degradation pathways and the meiotic cycle. Inhibition of MAPK pathway is associated with disruption in protein translation during crucial events for oocyte maturation, such as microtubule rearrangement and meiotic spindle assembly (Lefebvre et al., 2002; Zhang et al., 2015). Inherited cytoskeletal components, such as microtubule organizing centers (MTOCs), provide structural support during early embryonic development (Li and Albertini, 2013), highlighting the importance of mRNA translation mechanisms that operate during oocyte maturation for early development. Moreover, the somatic compartment mediates an important control of the translational process in oocytes, given that EGF pathway from granulosa and cumulus cells upregulates PI3K-Akt-mTOR signaling and TPX2 and DAZL protein levels in oocytes (Chen et al., 2013).

The control of these transcript stores is evolutionarily conserved and can be controlled by the length of the mRNA poly(A) tail, which determines the mRNA stability and can direct the transcript toward the translation or degradation machinery (Radford et al., 2008; Brook et al., 2009). The elongation (polyadenylation) and the shortening (deadenylation) of the poly(A) tail are mediated through the recognition and binding of specific sequences in 3’ untranslated regions (3’ UTR), referred as “cis” sequences, by “trans” RNA-binding proteins (RBP), and recruitment of polymerase or deadenylase complexes (McGrew and Ritcher, 1990; Piqué et al., 2008). The cytoplasmic mRNA polyadenylation relies on a hexanucleotide polyadenylation signal (AAUAAA) and a U-rich cytoplasmic polyadenylation element (CPE) localized on 3’ UTR. Cytoplasmic polyadenylation element binding proteins (CPEB) recognize and bind CPE, and function as a switch depending on its post-translational modification: repression (unphosphorylated) or promotion (phosphorylated) of poly(A) tail elongation (Hake and Richter, 1994; Walser and Lipshitz, 2011). Active CPEB enhances the polymerase activity of poly(A)-specific ribonuclease (PARN). Poly(A) tail elongation triggers the recruitment of poly(A) binding proteins and their interaction with the members of eukaryotic translation initiation complex eIF4E and eIF4G at 5’ m^7^G cap, creating a stable circular structure and recruiting 40S ribosomal subunit to initiate translation (Hake and Richter, 1994; Radford et al., 2008; Weill et al., 2012).

The selective and temporal maternal transcript degradation is also essential for oocyte competence and embryo development (Su et al., 2007), and deadenylation of the poly(A) tail regulates this process. ZFP36L2, a member of the ZFP36 (CCCH *tandem zinc finger proteins* ZFP36) or Tristetraprolin family, is involved in deadenylation of the poly(A) tail during oocyte maturation (Ramos, 2012; Dumdie et al., 2018). ZFP36 family members are known for suppressing tumors and regulating oncogenes, cytokines, growth factors, metabolic regulators, and immune response (Vogel et al., 2016; Suk et al., 2018; de Toeuf et al., 2018; Wu et al., 2021). Through its zinc finger domains, ZFP36L2 binds to specific sequences known as adenine- and uridine-rich elements (AREs) in the 3’ UTR, and recruits and interacts with CNOT7, a member of CCR4-NOT deadenylase complex (Fenger-Gron et al., 2005; Sha et al., 2018). Phosphorylation of serine and threonine residues in the C-terminal region can inhibit the activity of the members of the ZFP36 family, and these sites are conserved between ZFP36L1 and 2 (Clark et al., 2009). ZFP36 members can be phosphorylated downstream of many signaling pathways such as p38 MAPK, ERK, GSK3β, JNK, and cAMP-dependent protein kinases A, B, and C (for review, see Sanduja et al., 2011). Phosphorylation changes the ZFP36L2 binding site structure, which results in decreasing affinity to CNOT7 and triggering ZFP36L2 binding with 14-3-3 proteins, thus impairing its activity (Clement et al., 2011). *Zfp36l2* knockout or protein truncation in mice results in normal cycling and ovulation in females but developmental arrest at the time of embryonic genome activation, emphasizing the importance of mRNA degradation mechanisms for proper embryo development (Ramos, 2012; Dumdie et al., 2018).

The main meiotic events are modulated by signaling pathways that initiate on the somatic compartment of the follicle, thus, the supplementation of follicular factors to improve *in vitro* oocyte maturation has been the effort of several studies in the last years. *In vitro* maturation systems employing cyclic nucleotide modulators or somatic-produced factors, such as the natriuretic peptide precursor C (NPPC) and the EGF-like peptide amphiregulin (AREG), have been showing promising results in terms of oocyte and embryo quality and *in vitro* production (Franciosi et al., 2014; Soares et al., 2017; Xi et al., 2018). In this study, we first observed that the conventional IVM system altered the expression of *ZFP36L2*, an mRNA degradation-related gene, in *in vitro* matured bovine oocytes compared with *in vivo* counterparts. Thereafter, we tested the effects of addition of a pre-IVM step supplemented with NPPC and the adoption of a modified IVM media with AREG on the expression of a subset of genes associated with mRNA translation and decay pathways in bovine COCs. We also evaluated, particularly, transcript and protein ZFP36L2 levels in oocytes, and some of its target genes. Our results showed that NPPC and AREG supplementation during pre- and IVM steps massively upregulated genes from mRNA regulation pathways in cumulus cells throughout IVM, although oocytes were less susceptible to alterations. Moreover, although ZFP36L2 mRNA and protein levels had been affected by the IVM protocols, little effect was observed on its target transcripts. These data suggest that the mRNA translational and decay machinery of cumulus cells are more susceptible to supplementation of follicular factors during IVM. This fact may shed light on the development of IVM protocols that focus on improving the somatic compartment as a source of molecules to the oocyte.

## Methods

All reagents were purchased from Sigma Chemical Co. (St. Louis, MO, USA) unless otherwise stated. The study was approved by the Bioethical Committee of the FZEA – University of Sao Paulo, Pirassununga, SP, Brazil, protocol number 14.1.675.74.7. We adopted the International Guiding Principles for Biomedical Research Involving Animals (Society for the Study of Reproduction).

### Experimental design

In the first step of this study, which consisted of analysis of the expression of two genes related to translation (*CPEB1* and *CPEB4*) and two associated with mRNA decay (*ZFP36L2* and *CNOT7*), COCs were collected after removal from follicles (immature) or matured *in vivo* or *in vitro* (in conventional IVM medium).

In the second part, selected COCs were directly *in vitro* matured for 19h in control IVM (Cm group) or modified IVM (Mm group) supplemented with 100 ng/mL AREG and steroid hormones. The third group consisted of COCs pre-matured during 9h in a medium supplemented with 100 nM NPPC and steroid hormones, following modified IVM (Pm-Mm group) for 19h. Samples were collected at 0h, 9h, and 19h of IVM. COCs at the end of pre-IVM (Pm-0h) were compared with COCs freshly isolated from follicles (COC-0h). At 19h IVM, only COCs presenting the first polar body extruded (MII stage) were utilized for analysis.

### *In vivo*-matured oocyte recovery

The *in vivo*-matured oocytes were obtained according to the protocol described by Del Collado et al. (2017a). Thirty-three cyclic Nellore cows were submitted to synchronization of the follicular wave and superstimulation. Briefly, the protocol consisted of the administration of 0.5 mg cloprostenol (Sincrocio, Ouro Fino Saude Animal), follicular ablation associated with 2 mg benzoate estradiol (Sincrodiol, Ouro Fino Saude Animal), and intravaginal progesterone-releasing device implants (Sincrogest, Ouro Fino Saude Animal) on Day 0. For follicular superstimulation, 133 mg of FSH (Folltropin, Bioniche Animal Health Canada Inc. Belleville, ON, Canada) was administered in 8 decreasing doses every 12h, per cow. A 0.5 mg cloprostenol dose was administered at the time of the sixth FSH application, and the progesterone device was removed 12 h later. On day 8, 0.02 mg GnRH analog buserelin acetate (Sincroforte, Ouro Fino Saude Animal) was administered and *ovum pick-up* (OPU) was performed 25-26h later to recover expanded COCs, and after denudation, only oocytes showing the first polar body extruded (MII stage) were collected for analysis. A total of 12 different OPU sessions were performed.

### Immature oocyte recovery and selection for IVM

Ovaries were collected from a commercial slaughterhouse and transported to the laboratory in a bottle containing saline solution (0.9% NaCl) at 30°C. Ovaries were washed and kept in prewarmed saline solution during the aspiration using an 18-gauge needle attached to a 10 mL syringe. Cumulus-oocyte complexes aspirated from 2-8 mm diameter follicles were retrieved in centrifuged follicular fluid, and those containing two or more compact cumulus cell layers and homogeneous cytoplasm were selected and washed 3 times in washing medium, composed by TCM199 with Earle's salts, L‐glutamine, 25 mM HEPES, 50 µg/mL gentamicin, 0.2 mM sodium pyruvate, and 10 mg/mL fatty acid-free bovine serum albumin (FAF BSA).

### Pre-maturation (pre-IVM) culture

Selected COCs were submitted to pre-IVM (following protocol by Soares et al., 2017) in TCM199 with Earle’s salt, L-glutamine, 2.2 g/L sodium bicarbonate (GIBCO, cat. 11150-059), 4 mg/mL BSA, 50 µg/mL gentamicin, 0.2 mM sodium pyruvate, 100 nM NPPC, 10^-4^ IU/mL r-hFSH (Gonal-F, Merck, Darmstadt, Germany), 500 ng/mL oestradiol (E2758), 50 ng/mL progesterone (P8783) and 50 ng/mL androstenedione (A9630). COCs were pre-matured for 9h in four-well dishes containing 400 µL pre-IVM medium per well (up to 50 COCs/well), at 38.5°C, 5% CO_2_, and maximum humidity.

### *In vitro* maturation (IVM)

COCs were *in vitro* matured for 19h, according to the experimental group: (a) Control (Cm), in TCM199 with Earle’s salt, L-glutamine, 2.2 g/L sodium bicarbonate (GIBCO), 10% FCS (GIBCO), 0.5 µg/mL FSH (Folltropin; Ouro Fino Saude Animal, Cravinhos, Brazil). 50 mg/mL hCG (Vetecor; Ouro Fino Saude Animal), 50 µg/mL gentamicin and 0.2 mM sodium pyruvate; or (b) modified IVM (Mm; Soares et al. 2017 with modifications), in TCM199 with Earle’s salt, L-glutamine, 2.2 g/L sodium bicarbonate (GIBCO), 4 mg/mL FAF BSA, 50 µg/mL gentamicin, 0.2 mM sodium pyruvate, 10 ng/mL r-hIGF-1 (Invitrogen), 100 ng/mL AREG (A7080), 10^-2^ IU/mL r-hFSH (Gonal-F), 5 µg/mL oestradiol (E2758), and 150 ng/mL progesterone (P8783); (c) pre-matured COC were matured in modified media. COCs were incubated in four-well dishes containing 400 µL IVM media per well (up to 50 COCs/well) at 38.5°C, 5% CO_2_, and maximum humidity.

### Gene expression analysis in cumulus cells and oocytes at 0h, 9h and 19h of IVM

In order to evaluate the levels of target transcripts between (1) immature, *in vivo*, and *in vitro* matured oocytes and (2) cumulus cells and oocytes at 0h, 9h, and 19h of IVM, pools of 10 COCs per experimental group were retrieved from seven biological replicates and denuded by pipetting in 0.1% polyvinylpyrrolidone in PBS. At 19h IVM, COCs were individually denuded and pools contained samples (both CC and oocytes) at the MII stage. Cumulus cells were transferred into RNase/DNase-free 1.5 mL tubes and centrifuged twice at 250 x *g* for 5 min at room temperature. The supernatant was discarded and the tubes containing CC pellets were snap-frozen in liquid nitrogen. The completely denuded oocytes were transferred into RNase/DNase-free 1.5 mL tubes in minimum liquid volume and snap-frozen in liquid nitrogen. Frozen samples were stored at -80°C until analysis.

Total RNA was extracted with TRIzol reagent (Invitrogen) according to the manufacturer’s instructions, with the following modifications: after the addition of 1.33 µL GlycoBlue Coprecipitant (Thermo Fisher) to the aqueous phase, samples were subjected to centrifugation at 20,000 x g for 30 min. The supernatants were discarded and two centrifugations at 12,500 x *g* for 5 min with 1 mL 75% ethanol in ultrapure water were performed to reduce contaminations. The final RNA pellet was diluted in 10 µL of ultrapure water, and samples were treated with DNase I (Invitrogen) for genomic DNA elimination. Total RNA was quantified in the NanoDrop One^C^ system (Thermo Fisher Scientific). Samples were normalized and cDNAs were synthesized using the High Capacity cDNA Reverse Transcription Kit (Applied Biosystems) according to the manufacturer’s instructions.

For real-time quantitative PCR (RT-qPCR) in oocytes, 10 ng of cDNA was used per RT reaction. For CC samples, an 8x dilution in ultrapure water was necessary due to the number of target genes, resulting in 7.5 ng of cDNA per reaction. Reactions were performed using Power SYBR Green PCR Master Mix (Applied Biosystems) in the QuantStudio 6 Flex PCR System (Applied Biosystems), with the following conditions: 95°C for 10 min, 40 cycles of 95°C of 15 seg, and 60 seg at 60°C. The CT data were normalized by the subtraction of the geometric mean of five housekeeping genes (*ACTB*, *PPIA*, *GUSB*, *RPL15,* and *SDHA*) for CC and four (*ACTB*, *PPIA*, *GUSB,* and *RPL15*) for oocyte samples. The primers for target genes in cumulus (*EIF4A3, EIF4B, EIF4E, EIF4G2, PABPC1, PABPN1, PAIP1,* and *YWHAZ*) and in oocytes (*CPEB1, CPEB4, CNOT7, MAPKAPK2*, *ZFP36L2, KDM1B, KDM3B, KDM4B, KDM4C, KDM5A, KDM5B,* and *KDM5C*) were designed using NCBI and Ensembl sequences (Tables 1 and 2) and tested for efficiency before the analyses.

**Table 1:** Primer sequences for gene expression analyses in cumulus cells and oocytes, by RT-qPCR.

**Gene**	**Primer sequence**	**Reference**
***EIF4A3***	F: ATGAAGGAGTTCCGGTCAGG R: ACCTGATCGCCCAATTCTGT	ENSBTAG00000016023
***EIF4B***	F: ACGACTCCAGATCTGCACCTG R: TCTTCACCGTCAATGGCGAGA	ENSBTAG00000006883
***EIF4E***	F: TTAATGCCTGGCTGTGACTAC R: ACGATCGAGGTCACTTCGTCT	ENSBTAG00000009522
***EIF4G2***	F: CGTTTCAGTGCTTCTTCGGG R:CTGCGGAGTTGTCATCTCGT	ENSBTAG00000020308
***PABPC1***	F: CAAATACGCTGCGGGAGTTC R: AGAGCCGTTCACCCAACATT	ENSBTAG00000046358
***PABPN1***	F: TGGCCATCCGAAAGGGTTTG R: CCTCGGTCTGTTGTGCTGAT	ENSBTAG00000006884
***PAIP1***	F: GGGCCCCAGAACAAACGAG R: GGGTAGAATTCAGGGGCGTT	ENSBTAG00000020376
***CPEB1***	F: TCATGATCATTTGCCAGACTTCC R: AGTCTGAGTCCTGGGTGCTC	XM_027521170.1
***CPEB4***	F: GGTGTGTGCTATGCTGGGAT R: TGGCTTAACTTCCACCCGTT	XM_027520495.1
***CNOT7***	F: GCTCAGCGACACAAGTACATA R: GCATAGTGAGGGCACAAGGA	XM_027530133.1
***ZFP36L2***	F:CAGTTTCCAGCTCCGACCAT R: GGGATTTCTCCGTCTTGCAC	XM_027555128.1
***MAPKAPK2***	F: CATGCAATCGACGAAGGTCC R: CACTGGTCATCTCCTCCTTGAC	XM_027565933.1
***YWHAZ***	F: GCATCCCACAGACTATTTCC R:GCAAAGACAATGACAGACCA	ENSBTAG00000000236
***ACTB***	F: CAGCAGATGTGGATCAGCAAGC R: AACGCAGCTAACAGTCCGCC	ENSBTAG00000026199
***PPIA***	F: GGTCCTGGCATCTTGTCCAT R: TGCCATCCAACCACTCAGTCT	ENSBTAG00000012003
***SDHA***	F: AACCTGATGCTTTGTGCTCTGC R: TCGTCAACCCTCTCCTTGAAGT	ENSBTAG00000046019
***GUSB***	F: AGAGCGAGTACGGAGCAGATG R: AGCAGGCCTTTCTGGTACTCTTC	ENSBTAG00000000704
***RPL15***	F: CAAACGCCCAGTTCCTAAGG R: TCGAGCAAACTTGAGCTGGTT	ENSBTAG00000033080

**Table 2:** Primer sequences for ZFP36L2 target genes, evaluated by RT-qPCR in oocytes.

Gene	Primer sequence	Reference
***CCNE1***	F: GCTGCCAAACTTGAGGAAATCT R: GGACACAATGGTCAGGGGAC	XM_024978360.1
***FBXO43***	F: TGAAAACAAATTCTGAGGGGGC R: AACCTGGTGTCCTAGCCTGT	XM_010812178.3
***FBXO5***	F: GGAAGATGACAAGGTGGCATT R: CAAAGTTTTGGCGACAGTAATTC	NM_001082435.2
***KDM1B***	F: GCTCTTCAAGGAGCAGGAGG R: CTTCACGAAGCTGTAGGCCA	ENSBTAG00000014608
***KDM3B***	F: AACTGAGAGTGGTGAGATCAAGT R: TCCCCTCTATGCTCTCTCTC	ENSBTAG00000010063
***KDM4B***	F: TGCCGGAAACGGATGAAGAA R: TAGTAAAGCCCGTTGCGGTT	ENSBTAG00000015487
***KDM4C***	F: TTTCCCAAATCTCCCTGGGC R: GCTGGAGCAGTCTCTGGC	XM_027549308.1
***KDM5A***	F: GGTGTTTGAGCTTGTGCCTG R: TGTAACACGACTGACCCACG	ENSBTAG00000020472
***KDM5B***	F: GACGTGTGCCAGTTTTGGAC R: TCGAGGACACAGCACCTCT	ENSBTAG00000006175
***KDM5C***	F: ATCACTACCCCTGCCTGGAT R: CTGAGGTCCTGCGCAGATAG	ENSBTAG00000014943

### Immunostaining of ZFP36L2 in oocytes at 9h and 19h of *in vitro* maturation

Quantification of protein ZFP36L2 in oocytes at 9h and 19h of IVM were performed by immunostaining protocol according to Sangalli et al. (2022) in 39 denuded oocytes from Cm-9h, 44 Mm-9h, 49 Pm-Mm-9h, 27 Cm-MII, 38 Mm-MII, and 30 Pm-Mm-MII. Mouse embryonic fibroblasts were utilized as positive and negative controls at the first antibody efficiency test, and 5-10 denuded oocytes per group and per time point were utilized as negative control, with subtraction of primary antibody incubation. For this analysis, oocytes at 9h and 19h IVM (MII stage) were completely denuded from cumulus cells by pipetting and fixed in a 4% paraformaldehyde solution. Fixed oocytes were washed in 0.1% Triton X-100 and 1% BSA in PBS (washing buffer) for 10 min following permeabilization in 1% Triton X-100 in PBS for 30 min. After, oocytes were washed for 10 min and blocked for 1h in a blocking buffer containing 5% BSA and 10% goat serum in PBS. After blocking, oocytes were washed again for 10 min and incubated overnight at 4°C with mouse monoclonal ZFP36L2 antibody (Santa Cruz Biotechnology, SC365908, 1:500). After incubation oocytes were submitted to 3 washes of 10 min and 3 washes of 20 min, following incubation during 1h with goat anti-mouse Alexa Fluor 488 secondary antibody (Thermo Fisher, A11001, 1:500) at room temperature and protected from light. Then, oocytes were submitted to three washes of 10 min, incubated for 30 min in 10 µg/mL Hoechst 33342 (Thermo Fisher; 1:1000) in the washing solution, and then washed once for 20 min. Oocytes were mounted in glass slides in 20 µL ProLong™ Gold Antifade Mountant (Invitrogen) and covered with coverslips. Images were captured in 63x objective in Leica DMi8 Inverted Fluorescence Microscope with Thunder Imaging System, with excitation/emission wavelengths of 488 nm/496 nm for AF 488 and 352 nm/454 nm for Hoechst 33342. Fluorescence intensity for ZFP36L2 was measured by Fiji software (National Institutes of Health, Bethesda, MD), following the adoption of a two-step normalization process (CTCF1 and CTCF2), where CTCF (corrected total cell fluorescence) = integrated density - (oocyte area * mean fluorescence of background readings). In CTCF1, we subtracted the mean fluorescence from five background reads around the oocyte image, and in CTCF2 we utilized CTCF1 as integrated density and subtracted the mean fluorescence of negative controls oocytes.

### Statistical analysis

ZFP36L2 protein quantification and normalized CT data were tested for homogeneity and normality, and analyzed by one-way ANOVA followed by Tukey’s test for multiple comparisons using GraphPad Prism 8.0.1 (protein quantification) and SAS 8.2 (gene expression analysis). Means were considered significantly different at 5% level (p<0.05).

## Results

### Compared with the immature group, pre-IVM with NPPC upregulated genes related to mRNA translation and decay machinery in cumulus cells

**Figure 1:**
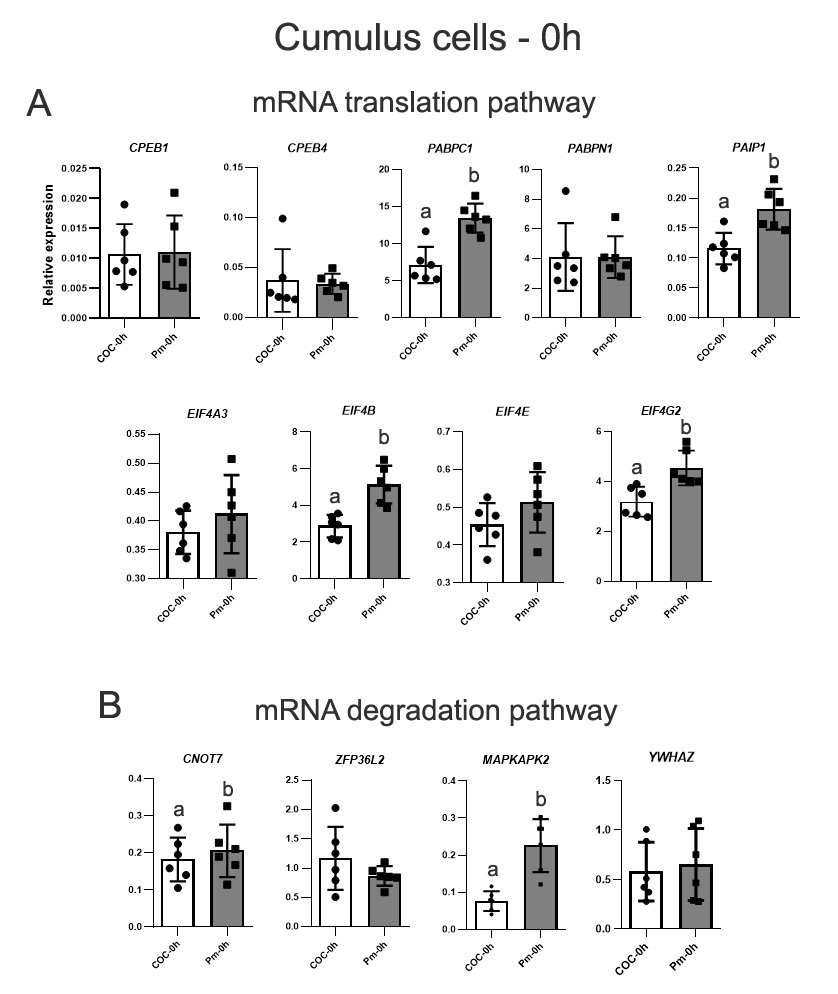
Relative expression of genes associated with mRNA translation (A) and degradation (B) pathways in cumulus cells*,* analyzed by RT-qPCR. *EIF4A3, EIF4B, EIF4E, EIF4G2, PABPC1, PABPN1, PAIP1*, *CPEB1*, *CPEB4*, *CNOT7*, *ZFP36L2*, *MAPKAPK2*, and *YWHAZ* levels were evaluated in immature (COC-0h) and pre-matured cumulus cells with NPPC supplementation (Pm-0h). CT mean from each gene was normalized by the geometric mean of housekeeping genes *ACTB*, *PPIA*, *SDHA*, *GUSB,* and *RPL15*. Different letters above bars in the same graph indicate differences (p<0.05). Values are presented as mean ± standard error of the mean.

Pre-IVM with NPPC for 9h induced increases in the expression of four genes related to the mRNA translation process in cumulus cells (Figure 1). *EIF4B* (fold-change 1.8, p<0.0001), *EIF4G2* (fc 1.4, p=0.0002), *PABPC1* (fc 1.9, p=0.0001) and *PAIP1* (fc 1.6, p=0.0003) were upregulated in pre-matured cumulus cells compared with immature control. Among the genes involved in the deadenylation and transcript degradation, only *CNOT7* (fc 1.15, p=0.0464) showed increased levels at the end of pre-IVM. *MAPKAPK2*, related to signaling pathways and which is a target for its involvement with ZFP36L2 inactivation, were upregulated by NPPC treatment (fc 2.9, p<0.0001), compared to oocytes freshly isolated from follicles (Figure 1).

### Modified IVM with AREG upregulated genes related to mRNA translation and decay machinery in cumulus cells throughout IVM

**Figure 2:**
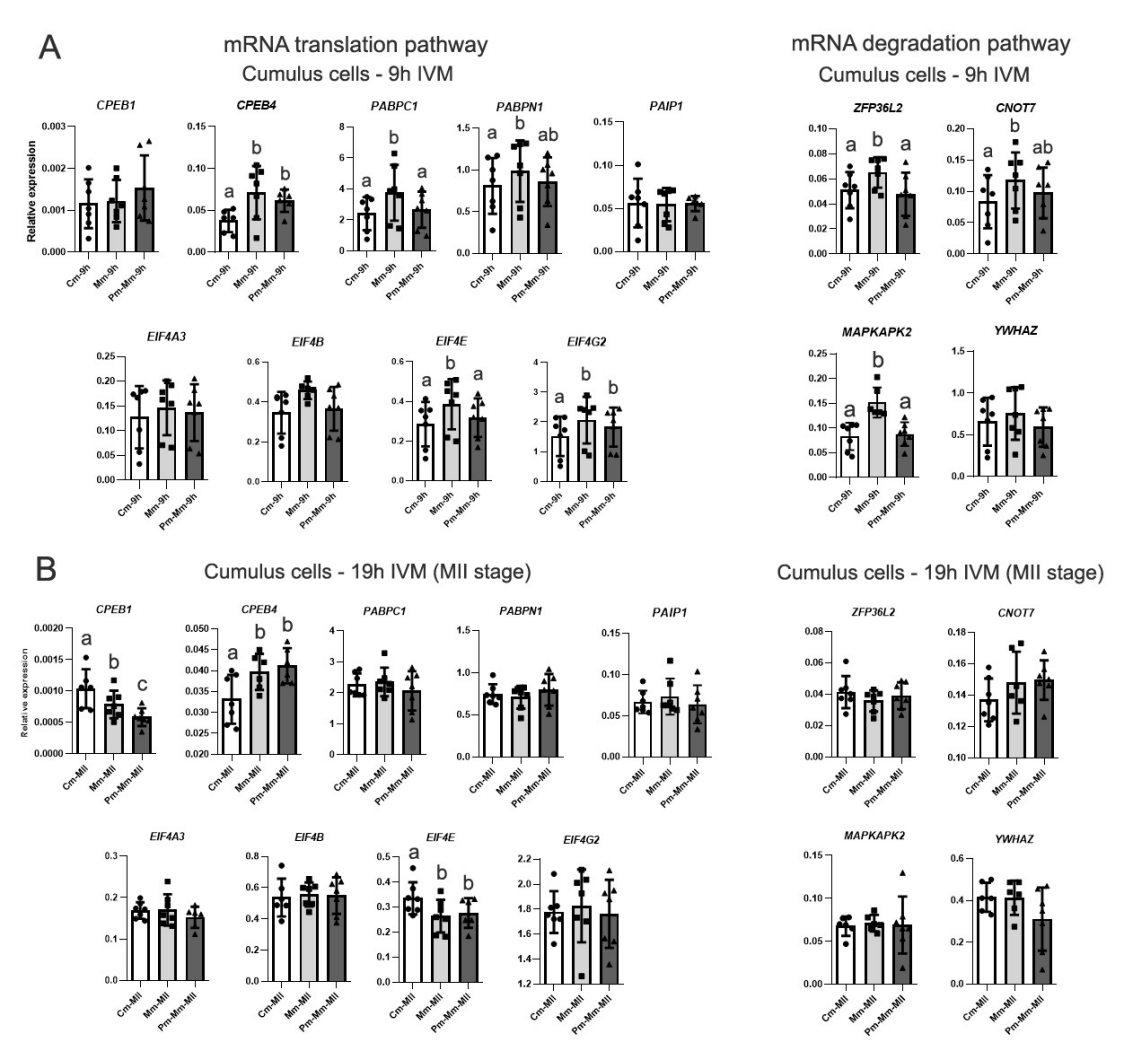
Relative expression of genes associated with mRNA translation and degradation pathways in cumulus cells*,* analyzed by RT-qPCR. *EIF4A3, EIF4B, EIF4E, EIF4G2, PABPC1, PABPN1, PAIP1*, *CPEB1*, *CPEB4*, *CNOT7*, *ZFP36L2*, *MAPKAPK2*, and *YWHAZ* levels were evaluated in cumulus cells from COCs cultured in control IVM (conventional IVM; Cm), modified IVM with AREG (Mm), and modified IVM preceded by pre-IVM with NPPC (Pm-Mm) at 9h of IVM (A), and at 19h of IVM in cells recovered from oocytes at MII stage (B). CT mean from each gene was normalized by the geometric mean of housekeeping genes *ACTB*, *PPIA*, *SDHA*, *GUSB,* and *RPL15*. Different letters above bars in the same graph indicate differences (p<0.05). Values are presented as mean ± standard error of the mean.

At 9h IVM, AREG-modified IVM induced increases in the expression of five genes related to translation and three genes related to transcript degradation in cumulus cells (Figure 2A). *CPEB4*, which encodes a polyadenylation regulator protein, was upregulated in Mm-9h (fc 1.8) and Pm-Mm-9h (fc 1.6) (p<0.0001) compared with IVM control. The same was observed for *EIF4G2* (p=0.0009; Mm-9h fc 1.4; Pm-Mm-9h fc 1.2), a gene that produces a non-functional variant of EIF4G. *EIF4E* was upregulated by AREG supplementation (fc 1.4, p=0.0014), but the adoption of the pre-IVM step inhibited this effect (Figure 2). From genes related to mRNA decay, *ZFP36L2* (fc 1.3, p=0.0022) and *MAPKAPK2* (fc 1.8, p<0.0001) were upregulated only in cumulus oocytes directly matured in modified medium, compared to the control and pre-IVM groups. *CNOT7* levels were higher (fc 1.4, p=0.0022) in Mm-9h compared with control (Cm). This expression pattern was also observed in *PABPN1* (fc 1.2, p=0.0208), while *PABPC1* was upregulated (fc 1.6, p<0.0001) in cumulus cells directly matured with AREG (Figure 2A).

Differences in gene expression were also observed in cumulus from MII oocytes (Figure 2B). At 19h of IVM, only three genes related to mRNA translation showed differences in expression between IVM protocols. *CPEB1* was differentially expressed (p=0.005) between all groups, showing higher levels in control IVM, intermediate in modified IVM (fc 0.8), and lower levels in the group submitted to pre-IVM (fc 0.6). *CPEB4* was upregulated (p=0.0019) in cumulus matured with AREG (Mm-MII fc 1.3; Pm-Mm-MII fc 1.2) compared to Cm-MII. AREG supplementation downregulated (p=0.0045, fc 0.8) *EIF4E* expression compared to control IVM (Figure 2B).

### *ZFP36L2* was downregulated in in vitro matured oocytes compared with in vivo

**Figure 3:**
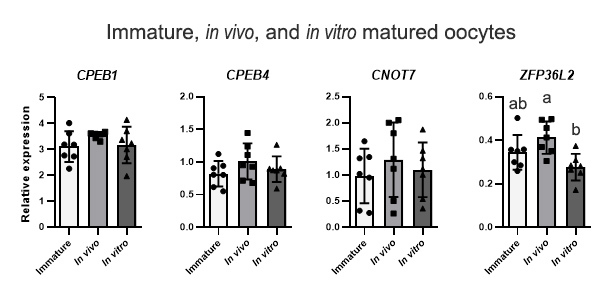
Relative expression of genes associated with mRNA translation and degradation pathways in denuded oocytes*,* analyzed by RT-qPCR. *CPEB1*, *CPEB4*, *CNOT7,* and *ZFP36L2* relative mRNA levels were analyzed in immature, *in vivo*, and *in vitro* (conventional IVM) matured oocytes. CT mean from each gene was normalized by the geometric mean of housekeeping genes *ACTB*, *PPIA*, *GUSB,* and *RPL15*. Different letters above bars in the same graph indicate differences (p<0.05). Values are presented as mean ± standard error of the mean.

In order to verify if the IVM system affected gene expression of two genes related to translation (*CPEB1* and *CPEB4*) and two genes related to the transcript decay process (*CNOT7* and *ZFP36L2*) we performed RT-qPCR in immature, *in vivo* matured, and *in vitro* matured oocytes. From these four genes, only *ZFP36L2* was affected by IVM, showing lower expression in *in vitro* matured (fc 0.67, p=0.0083) compared with *in vivo* counterparts (Figure 3).

### Modified media with AREG downregulated *ZFP36L2* expression in oocytes throughout IVM

Since IVM affects *ZFP36L2* expression in oocytes, we aimed to compare its expression between the three different IVM protocols. *CNOT7*, *MAPKAPK2,* and *YWHAZ* expressions were also evaluated, due to their link with the ZFP36L2 activity, as well as *CPEB1* and *CPEB4* transcript levels, which are proteins that regulate the polyadenylation of the *ZFP36L2* mRNA. As *ZFP36L2* expressions were not different between immature and both (*in vivo* and *in vitro*) matured oocytes nor between the immature (COC-0h) and pre-matured oocytes (Pm-0h) (data not shown), we performed analyses only at 9h and 19h (MII stage) of IVM.

**Figure 4:**
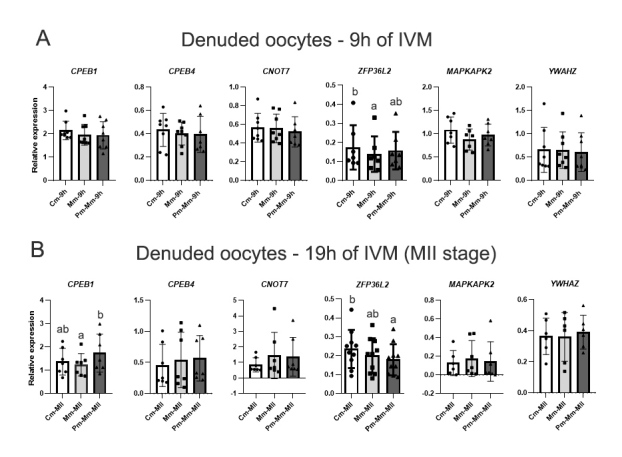
Relative expression of genes associated with mRNA translation and degradation pathways in denuded oocytes*,* analyzed by RT-qPCR. *CPEB1*, *CPEB4*, *CNOT7*, *ZFP36L2*, *MAPKAPK2*, and *YWHAZ* levels were evaluated in oocytes from control IVM (conventional IVM; Cm), modified IVM with AREG (Mm), and modified IVM preceded by a pre-IVM with NPPC (Pm-Mm), in oocytes at 9h (A) and 19h of IVM (B; oocytes at MII stage). CT mean from each gene was normalized by the geometric mean of housekeeping genes *ACTB*, *PPIA*, *GUSB,* and *RPL15*. Different letters above bars in the same graph indicate differences (p<0.05). Values are presented as mean ± standard error of the mean.

At 9h of IVM, *ZFP36L2* was downregulated (fc 0.8, p=0.026) in Mm-9h compared with Cm-9h (Figure 4A). In MII oocytes, *ZFP36L2* was downregulated (fc 0.7, p=0.0557) in Pm-Mm compared with control IVM. The addition of pre-IVM step upregulated *CPEB1* (fc 1.4, p=0.0132) in MII oocytes compared with those directed matured with AREG (Figure 4B).

### From the ZFP36L2 targets, only *KDM4C* levels were altered in oocytes by modified IVM

**Figure 5:**
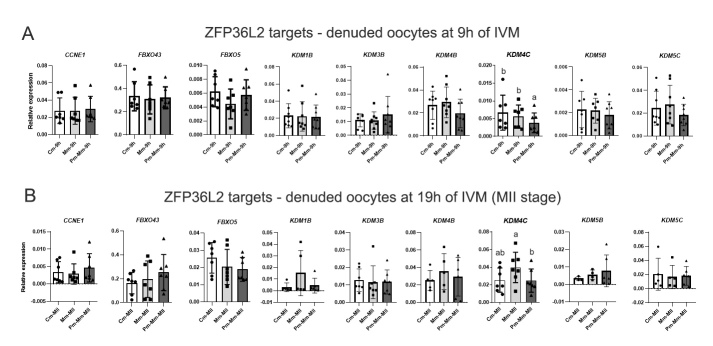
Relative expression of target genes of the ZFP36L2 protein, analyzed by RT-qPCR. *KDM1B*, *KDM3B*, *KDM4B*, *KDM4C*, *KDM5A, KDM5B*, and *KDM5C* mRNA levels were evaluated in denuded oocytes from control IVM (conventional IVM; Cm), modified IVM with AREG (Mm), and modified IVM preceded by pre-IVM with NPPC (Pm-Mm) at 9h IVM (A), and at 19h IVM in oocytes at MII stage (B). CT mean from each gene was normalized by the geometric mean of housekeeping genes *ACTB*, *PPIA*, *GUSB,* and *RPL15*. Different letters above bars in the same graph indicate differences (p<0.05). Values are presented as mean ± standard error of the mean.

Given that we observed differences in *ZFP36L2* expression in oocytes during IVM and in expressions of genes associated with ZFP36L2 activity in cumulus, we evaluated expression levels of some ZFP36L2- mRNA targets in oocytes at 9h and 19h of IVM. From 10 target genes chosen for evaluation, three (*CCNE1*, *FBXO43,* and *FBXO5*) are linked to cell cycle regulation, and seven (*KDM1B*, *KDM3B*, *KDM4B*, *KDM4C*, *KDM5A*, *KDM5B*, *KDM5C*) are histone demethylases with important roles in the chromatin condensation process. Among the 10 genes, only *KDM4C* levels were affected by IVM type at both time points. At 9h, the addition of the pre-IVM step resulted in downregulation (fc 0.6, p=0.0078) in *KDM4C* compared with Cm and Mm (Figure 5A). At 19h, higher levels of *KDM4C* transcript were observed in MII oocytes matured with AREG (fc 1.6, p=0.0058) compared with those also matured with ARE supplementation but previously treated with NPPC (Figure 5B).

### Modified media with AREG decreased ZFP36L2 protein levels in oocytes at 9h and 19h IVM

**Figure 6:**
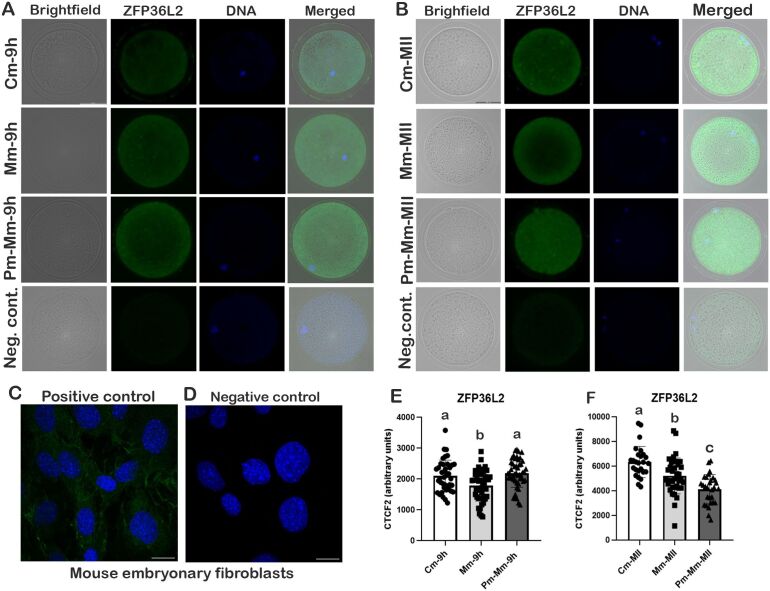
Representative images of oocytes immunostained for ZFP36L2 (green) and DNA (blue) at 9h IVM (A) and 19h (B) from control IVM (conventional IVM; Cm), modified IVM supplemented with AREG (Mm) and modified IVM preceded by pre-IVM with NPPC (Pm-Mm). Images were acquired in Leica DMi8 Inverted Fluorescence Microscope, in 63x magnification (scale bar: 50 µm). Mouse embryonic fibroblasts were used as positive (C) and negative control (D). ZFP36L2 protein levels in oocytes at 9h (E) and 19h (MII stage, (F) from Cm, Mm, and Pm-Mm groups. Values are presented as mean ± standard error of the mean. Different letters above bars in the same graph indicate differences (p<0.05).

After we observed that AREG altered *ZFP36L2* and *KDM4C* expressions at 9h and 19h of IVM, we aimed to quantify ZFP36L2 protein levels in these two time points. At 9h of IVM, a lower (fc 0.85, p=0.00035) ZFP36L2 protein level was observed in oocytes in IVM supplemented with AREG (Mm) compared to the other two groups (Cm and Pm-Mm) (Figure 6A and E), in a similar pattern as observed in *ZFP36L2* mRNA expression (Figure 4A). In MII oocytes, ZFP36L2 protein levels were different (p<0.0001) between all groups, with higher levels in the control group, intermediate in modified IVM (fc 0.82), and lower levels in oocytes submitted to pre-IVM (fc 0.65) (Figure 6B and F). Also at this time point, the ZFP36L2 protein expression pattern was similar to its mRNA expression (Figure 4B).

## Discussion

Our findings demonstrated that *in vitro* supplementation of somatic cell-produced factors, such as NPPC and AREG, can modulate the expression of several genes related to mRNA regulation in cumulus cells, and genes involved with the remodeling of maternal transcript stores in oocytes. These data suggest that meiotic modulator protocols could intensify translational processes, which possibly increase protein production and may improve oocyte competence *in vitr*o. NPPC has been shown to promote increases in cumulus cells cGMP levels and to prolong cumulus-oocyte communication via gap junctions (Franciosi et al., 2014; Jia and Wang, 2020). Studies have demonstrated that cumulus cells can supply the oocyte, through transzonal projections, with RNA and proteins (Macaulay et al., 2014; Del Collado et al., 2017b). Since the final growth of the oocyte involves chromatin condensation and transcriptional silencing, cumulus metabolic and molecular supply are necessary to modulate cytoplasmic and nuclear events in the gamete, including activation of transcriptional machinery and remodeling of maternal mRNA stores (Clarke, 2012; Conti et al., 2016). Thus, the increments in protein synthesis the in somatic compartment may be beneficial, as a source of micro and macromolecules, to the final phase of oocyte growth.

The NPPC-mediated pre-IVM and modified IVM with AREG resulted in the upregulation of a subset of genes associated with the mRNA translation and degradation pathways in cumulus cells. Genes related to members of the eukaryotic translation initiation factor (eIF) complex (*EIF4B*, *EIF4E*, *EIF4G2*), and associated poly(A) binding proteins *PABPC1*, *PABPN1*, and *PAIP1* showed increased expressions in NPPC and AREG groups at 0h and 9h of IVM. The cyclic nucleotides modulation promoted by these protocols could be the explanation for this massive gene expression mobilization, given that cGMP and cAMP can increase gene expression in several cell types, including cumulus (for a review, see Pilz and Casteel, 2003; Campen et al., 2016). The increase of cGMP also sustains higher cAMP levels in the cumulus, which can lead to the activation of protein kinase A (PKA). PKA, in turn, can trigger the upregulation of epidermal growth factor-like peptides amphiregulin (AREG), epiregulin (EREG), and betacellulin (BTC) in cumulus cells and transactivation of EGF receptor (EGFR), which leads to activation of extracellular regulated kinases 1 and 2 (ERK1/2) cascade (Su et al., 2007; Conti et al., 2006). Among ERK1/2 substrates, p90 ribosomal S6 kinase (RSK) can induce translational initiation through phosphorylation of eukaryotic translation initiation factor 4B (eIF4B) (Sonenberg and Hinnebusch, 2009; Andreou et al., 2017). Our data show a NPPC-induced upregulation of *EIF4B*, *EIF4G2*, *PABPC1,* and *PAIP1* in cumulus cells. All of these genes are associated with modulation of 5’ m^7^G cap-mediated translation. EIF4B is a co-factor that interacts with eIF4A and eIF4G to form auxiliary bridges between RNA and 40S ribosomal subunit (Méthot et al., 1996). *EIF4G2* encodes an eIF4G1 homolog that participates in non-canonical translation pathways due to the lack of binding domains for eIF4E and PABPs (Liu et al., 2023). Poly(A) binding protein cytoplasmic 1 (PABPC1), even as its nuclear homolog PABPN1, is found in both oocytes and embryos, and it seems to be downregulated in GV stage mouse oocytes after hormonal superovulation (Siemer et al., 2009; Ozturk et al., 2016). Poly(A) binding protein-interacting 1 (PAIP1) mediates the regulation of the PABP interaction with eIF4A (Craig et al., 1998).

*CNOT7* and *MAPKAPK2* expressions were upregulated in pre-matured and AREG-matured cumulus cells. The CCR4-NOT transcription complex subunit 7 (CNOT7), also known as CAF1, is one of the catalytic subunits of carbon catabolic repressor protein 4 (CCR4)-NOT deadenylase complex, the major deadenylase complex in eukaryotes and that promotes shortening of poly(A) tail, thus inhibiting transcript translation (Collart, 2016; Yi et al., 2018). *MAPKAPK2* gene encodes the p38 mitogen-activated protein kinase (MAPK)-activated kinase 2 (MK2), which is a kinase downstream of p38 MAPK signaling (Huot et al., 1995). FSH-mediated increases in PKA activity trigger p38 MAPK signaling, which in turn phosphorylates and activates MK2 (Yu et al., 2005; Yen et al., 2014). Activated MK2 can phosphorylate and inactivate ZFP36 members in many tissues, leading to ARE-mRNA stabilization (Chrestensen et al., 2004; Stoecklin et al., 2004; Clement et al., 2011). The inhibition of the ZFP36L2 activity is mediated by a structural change in the binding site caused by the phosphorylation in 493 and 495 serine residues in C-terminal region (Adachi et al., 2014). This reduces its affinity to CNOT7 and favors the binding of 14-3-3 proteins, resulting in an inactive complex (Chrestensen et al., 2004; Marchese et al., 2010; Clement et al., 2011). These findings suggest that the activation of cAMP-mediated signaling during pre-IVM could be downregulating mRNA decay pathways in the somatic compartment.

At 9h of IVM, our findings showed upregulation of several genes related to mRNA translation and decay in cumulus from COC directly matured with AREG compared with control IVM. The upregulation of *ZFP36L2*, *MAPKAPK2, PABPC1,* and *EIF4E* expression only in the Mm group suggests that pre-IVM with NPPC could have abbreviated these upregulations in a certain way. In mice, LH surge-induced *Zfp36* upregulated in granulosa cells is a mechanism mediated by activation of the EGFR-ERK1/2 pathway (Xi et al., 2021). Since AREG is an upstream activator of ERK1/2, it could explain the overexpression of *ZFP36L2* in cumulus from the Mm group. However, this effect has not been observed in Pm-Mm. Perhaps the lower *ZFP36L2* transcript levels in the latter did not reflect a lower gene expression, but a higher translation activity supported by the more expressed translation machinery factors during pre-IVM. It remains to be elucidated for further studies. The upregulation of *CNOT7* in cumulus could suggest that mRNA decay processes seem to be enhanced by the AREG-directed *in vitro* maturation. However, despite the *ZFP36L2* upregulation, *MAPKAPK2* was also upregulated cumulus in this group. ZFP36 family members are known to auto-regulate their expression through ARE sequences in their own 3’ UTR (Brooks et al., 2004). Thus, we do not know if the higher *ZFP36L2* transcript levels in directly ARE-matured cumulus cells are due to higher gene expression or lower transcript decay, given the possibility of decreased ZFP36L2 activity mediated by MK2-phosphorylation.

In cumulus from MII oocytes, we observed fewer genes differently expressed between control and treated groups. Three genes (*CPEB1, CPEB4,* and *EIF4E*) were differently expressed, albeit in a different fashion, by the addition of NPPC and AREG to IVM system. The expression patterns of *CPEB1* and *CPEB4* showed the opposite pattern. We could speculate that the alternation of activity between these proteins that is observed in oocytes during maturation (Igea and Méndez, 2010) could also be performed in cumulus cells, since the polyadenylation process during the mitotic cell cycle is also governed by both CPEB1 and CPEB4 (Novoa et al., 2010). The downregulation of *CPEB1* and *EIF4E* in cumulus from MII oocytes matured with AREG does not necessarily indicate decreases in translational activity, given that *CPEB4* was upregulated by this media and no differences were observed in the expressions from the other genes.

Our data showed that gene expression in oocytes was less susceptible to changes mediated by IVM strategies, albeit they subtly exist. In the first experiment of our study, we analyzed the expression levels of two genes related to translation (*CPEB1* and *CPEB4*) and two associated with the mRNA deadenylation pathway (*CNOT7* and *ZFP36L2*) in immature, *in vivo* matured, and *in vitro* matured oocytes. *In vitro* maturation resulted in downregulation of *ZFP36L2* in oocytes, compared with the *in vivo* counterparts. Although the superstimulation protocol used in our study to produce *in vivo* matured oocytes might not reflect the natural ovulation conditions, several studies have used similar protocols in order to compare oocytes matured *in vivo* and *in vitro* (Katz-Jaffe et al., 2009; Tesfaye et al., 2009; Del Collado et al., 2017a; Camargo et al., 2019). A study comparing gene expression in oocytes recovered from the same donors and matured *in vivo* (after FSH stimulation) and *in vitro* showed that IVM downregulated the levels of several polyadenylated mRNAs associated with developmental competence (Camargo et al., 2019). Moreover, FSH stimulation resulted in upregulation of transcripts related to mRNA translation and cell cycle in MII oocytes in comparison with naturally matured ones (Chu et al., 2012). Since our results showed a difference only in *ZFP36L2* levels between *in vivo* and *in vitro* matured oocytes, we could infer that this downregulation must have been influenced by IVM instead of the FSH stimulation.

In order to evaluate if a refined IVM protocol could improve *ZFP36L2* expression in oocytes, we tested the effects of a modified IVM with AREG with or without the addition of pre-IVM step with NPPC on transcript levels of *ZFP36L2*, *CNOT7*, *MAPKAPK2*, and *YWHAZ* (genes related to modulation of ZFP36L2 activity), and *CPEB1* and *CPEB4*, related to translational machinery, given the importance of these pathways to oocyte competence and early development (Mourot et al., 2006;; Graf et al., 2014). Unexpectedly, the addition of AREG downregulated *ZFP36L2* in oocytes at 9h of IVM, and this downregulation was pronounced in pre-matured MII oocytes at 19h of IVM. The same patterns of the *ZFP36L2* expression at 9h and 19h of IVM were observed in ZFP36L2 protein levels. Our data also showed that the only ZFP36L2 transcript target affected by IVM protocols was *KDM4C*, at both 9h and 19h of IVM. Collectively, these data highlight a probably, albeit subtle negative effect of the ERK1/2 activation in cumulus on the maternal transcript decay pathway.

In many cell types and tissues, ZFP36L2 is involved in the regulation of inflammatory and immune responses and it has been shown to regulate the development of tumors through the activation of the decay of cell cycle regulators (Vogel et al., 2016; Suk et al., 2018; de Toeuf et al., 2018; Wu et al., 2021). During meiotic progression, the ZFP36L2 targets include cyclins, early mitotic inhibitors 1 and 2 (Emi1 or FBXO5, and Emi2 or FBXO43), and several histone H4K3 and H4K9 demethylases that participate on the chromatin remodeling and transcriptional silencing events (Belloc and Mendez, 2008; Dumdie et al., 2018). The impairment of ZFP36L2 activity and thus the overexpression of its targets during meiosis result in defects in spindle morphology, chromosome alignment, and development arrest at embryonic genome activation (Ramos et al., 2004; Ramos, 2012; Ball et al., 2014; Liu et al., 2016; Zheng et al., 2022). However, the majority of the studies evaluating ZFP36L2 activity during oocyte and embryo development are based on genetic knockout or protein truncation, which leads to a total loss-of-function effect. Here, we have brought insights into a subtle effect caused by the inclusion of physiological modulators, NPPC and AREG, on an IVM protocol. It is possible that such an effect would not result in dramatic impairments in competence, since we have observed that just one from ten target genes of ZFP36L2 was upregulated in matured oocytes from the same group in which ZFP36L2 mRNA and protein levels were downregulated. However, given the susceptibility of ZFP36L2 to be modulated by ERK1/2/MAPK pathways (Adachi et al., 2014; Wang et al., 2015) further studies must be carried out to evaluate the impact of these ZFP36L2 mRNA and protein downregulations in meiosis and early developmental, due to the relevance of the adequate maternal transcript decay for embryonic genome activation (Sha et al., 2020a; Sha et al., 2020b).

In addition, *CPEB1* was upregulated in pre-matured MII oocytes, in a different fashion than those observed in cumulus cells at 19h of IVM. Both CPEB1 and CPEB4 alternate their action temporally during oocyte maturation, in an “early” and “late” waves of transcript polyadenylation. These waves are required for the temporal translation of key proteins to meiotic processes (Igea and Méndez, 2010). Although CPEB1 phosphorylation by ERK2 is not critical for the polyadenylation activity, it seems that CPEB4 requires ERK2- or Cdk1-mediated phosphorylation to be activated during oocyte maturation (Guillén-Boixet et al., 2016). The differences observed in *CPEB1* and *CPEB4* in both cumulus and oocytes in our study remain to be further investigated, since CPEB1-mediated disruption on the translational pathway was diagnosed in aging mice and associated with decreased oocyte quality (Takahashi et al., 2023).

## Conclusions

Our data showed that supplementation of NPPC in a pre-IVM step and AREG during modified IVM system promoted a massive upregulation, in cumulus cells, of genes associated with the mRNA translation and degradation programs, probably due to increases in ERK1/2 signaling pathways. These effects were less pronounced in oocytes, and although AREG and NPPC treatments have affected transcript and protein levels of the RNA-binding protein ZFP36L2, an mRNA-decay mediator, the broad expression of its target genes was not altered in oocytes during IVM, with the exception of *KDM4C*. Given the importance of cumulus cells as a source of micro and macromolecules for the oocyte, these findings collectively raise questions about the effects of meiotic modulation protocols on processes that could impact oocyte competence and early development.
